# Seebeck-voltage-triggered self-biased photoelectrochemical water splitting using HfO_x_/SiO_x_ bi-layer protected Si photocathodes

**DOI:** 10.1038/s41598-019-45672-4

**Published:** 2019-06-24

**Authors:** Jin-Young Jung, Dae Woong Kim, Dong-Hyung Kim, Tae Joo Park, Ralf B. Wehrspohn, Jung-Ho Lee

**Affiliations:** 1 Department of Materials and Chemical Engineering, Hanyang University, 55 Hanyangdaehak-ro, Sangnok-gu, Ansan, Kyeonggi-do, 15588 Republic of Korea; 2 Institute of Physics, Martin-Luther-Universität Halle-Wittenberg, Halle, Germany; 3 Fraunhofer Institute for Microstructure of Materials and Systems IMWS Walter-Hülse-Strasse 1, D06120 Halle, Germany

**Keywords:** Devices for energy harvesting, Electronic properties and materials

## Abstract

The use of a photoelectrochemical device is an efficient method of converting solar energy into hydrogen fuel via water splitting reactions. One of the best photoelectrode materials is Si, which absorbs a broad wavelength range of incident light and produces a high photocurrent level (~44 mA·cm^−2^). However, the maximum photovoltage that can be generated in single-junction Si devices (~0.75 V) is much lower than the voltage required for a water splitting reaction (>1.6 V). In addition, the Si surface is electrochemically oxidized or reduced when it comes into direct contact with the aqueous electrolyte. Here, we propose the hybridization of the photoelectrochemical device with a thermoelectric device, where the Seebeck voltage generated by the thermal energy triggers the self-biased water splitting reaction without compromising the photocurrent level at 42 mA cm^−2^. In this hybrid device p-Si, where the surface is protected by HfO_x_/SiO_x_ bilayers, is used as a photocathode. The HfO_x_ exhibits high corrosion resistance and protection ability, thereby ensuring stability. On applying the Seebeck voltage, the tunneling barrier of HfO_x_ is placed at a negligible energy level in the electron transfer from Si to the electrolyte, showing charge transfer kinetics independent of the HfO_x_ thickness. These findings serve as a proof-of-concept of the stable and high-efficiency production of hydrogen fuel by the photoelectrochemical-thermoelectric hybrid devices.

## Introduction

Solar water-splitting systems that directly convert solar energy into storable and transportable hydrogen fuel are a promising approach to sustainably providing cost-effective renewable energy^1–5^. In order to realize self-biased solar water splitting, intensive efforts have been made in the past decades to exploit designed prototype systems including photovoltaic-electrolysis combination cells^6–9^, tandem photoelectrochemical (PEC) cells using the dual-semiconductor light absorber^10–14^, and colloid-based “Z-scheme” photocatalytic cells^15–25^. These systems are normally better suited to generating an output voltage higher than the sum of thermodynamic potential (1.23 V) and kinetic overpotential (>0.3 V), which is required for a practical water-splitting reaction. However, it compromises the photocurrent level because a tradeoff relationship exists between the photovoltage and photocurrent in systems that operate based on the photovoltaic effect. This fundamental obstacle has limited the solar-to-hydrogen efficiency.

As a new approach to overcoming the aforementioned limitation, recently, the hybridization of the PEC cell with other energy-harvesting devices such as thermoelectric (TE) and piezoelectric generators—which generate an output voltage through the conversion of thermal and mechanical energy resources, respectively—has been proposed^26–28^. In particular, the PEC-TE hybrid device is expected to be a commercially viable approach because it can be operated with absorbed solar energy in the form of photons (using the PEC component) and phonons (using the TE component)^26,29^. The PEC-TE hybrid system—in which the PEC device is electrically connected with the TE device in series—could provide an additional output Seebeck voltage (V_TE_) as a result of a thermal gradient in the TE in order to overcome the potential barrier required for the water-splitting reaction. In this hybrid system, the overall current is determined by the high photocurrent level of the PEC cell (i.e., the TE is only used as a voltage booster). This feature of the PEC-TE hybrid system can decouple the tradeoff relation between the voltage and current, thus maximizing the H_2_ power generation (P_Max_).

Various light-absorbing semiconductors including narrow bandgap (Si, InP, and GaAs) and wide bandgap (TiO_x_, CuO_x_, FeO_x_, and BiVO_x_) materials have been used as photoelectrodes of PEC cell^30^. In the PEC-TE hybrid systems, because the output Seebeck voltage of the TE can further applied to drive the water splitting reaction, the narrow bandgap semiconductors with a higher photocurrent relative to the wide bandgap semiconductors are effective in maximizing the H_2_ power generation. Thus, Si with comparatively narrow bandgap (1.1 eV) is capable of generating a high photocurrent of ~44 mA·cm^−2^ via harnessing a large portion of the solar spectrum and is a suitable material as a light-absorbing semiconductor photoelectrode^31^. However, the poor corrosion-resistance of Si is an inherent drawback in terms of the long-term stable operation of the PEC reaction^32^. One suitable strategy for offsetting this drawback is to use a high-atomic-density insulating material as a protective layer that would enable the complete blocking of the electrolyte permeation^33–37^. In general, a high-atomic-density insulator of significant thickness provides excellent protection ability but results in an increase in the transfer resistance of the light-induced charge carriers owing to the tunneling barrier of the insulator^38^. Therefore, for Si PEC devices, it is of paramount importance to design a protection layer that guarantees both long-term stability and facile charge-transfer kinetics.

In this study, we present a PEC-TE hybrid system with a HfO_x_/SiO_x_ bilayer protected Si photocathode to ensure its long-term stability without the deterioration of the charge-transfer kinetics. The PEC-TE hybrid device based on an antireflective Si nanostructure photocathode enables the self-biased PEC water-splitting reaction at a high photocurrent level of ~42 mA·cm^−2^, which has a maximum H_2_ power generation of 55 mW·cm^−2^. The HfO_x_, which has strong corrosion resistance in an acidic electrolyte and a relatively higher atomic density (9.68 g·cm^−3^) than other dielectric materials such as SiO_x_ (2.65), AlO_x_ (3.95), and TiO_x_ (4.23), physically prevents the permeation of the electrolyte into the Si surface, thereby resulting in long-term stable PEC operation for 200 h. Moreover, on applying V_TE_, the electrical tunneling barrier of HfO_x_ becomes irrelevant to the charge transport because the potential drop caused by the application of V_TE_ mainly occurs in the SiO_x_ layer, which induces the tunneling barrier of HfO_x_ to be located at a level lower than the conduction band of Si. Therefore, V_TE_ is not only applicable for driving a water-splitting reaction but also for reducing the kinetic overpotential required for charge transfer.

## Results

**Figure 1:**
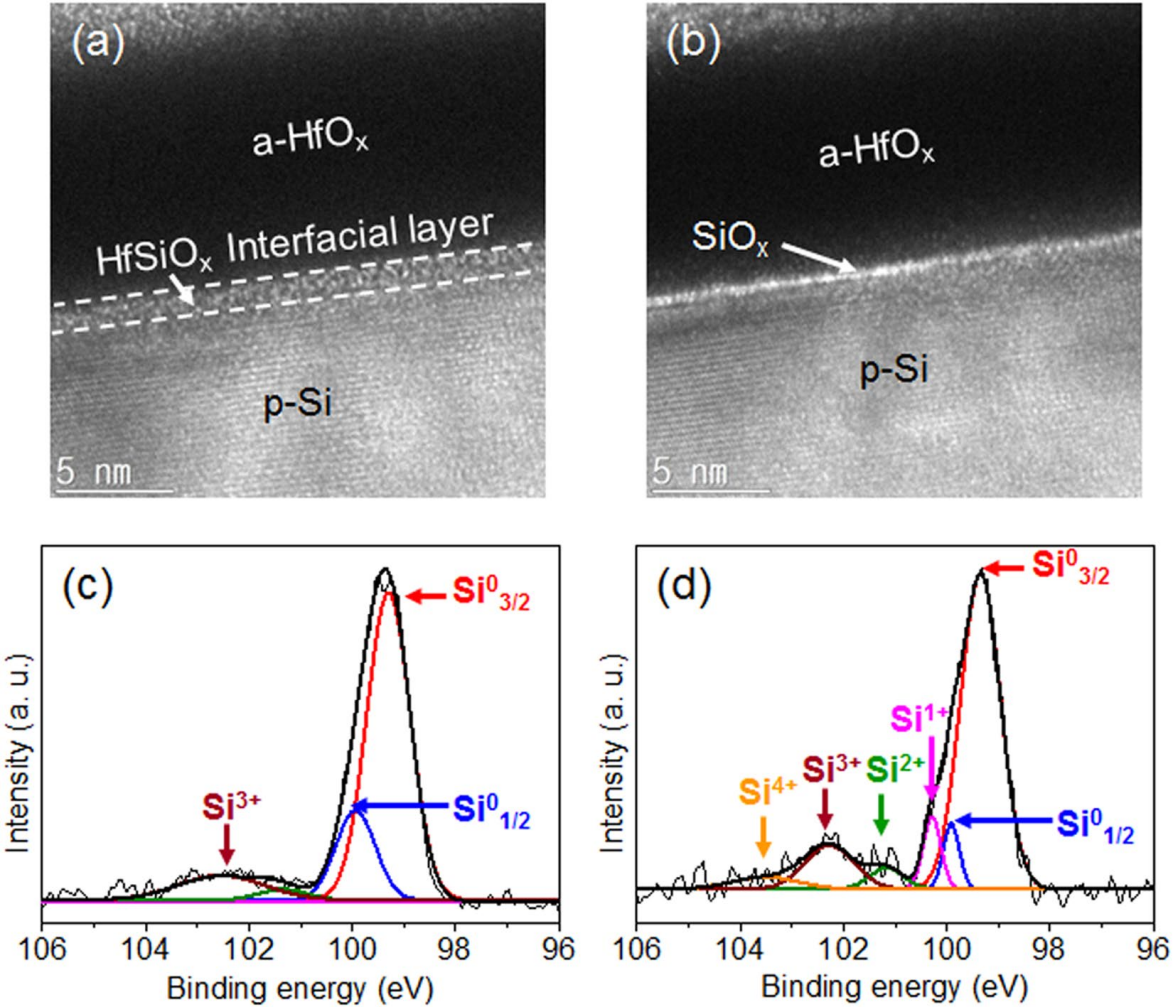
Formation of a-HfO_x_/SiO_x_ bilayer protected p-Si photocathode. TEM images and XPS spectra for the a-HfO_x_ deposited on (**a**,**c**) H-terminated and (**b**,**d**) SiO_x_-grown Si wafers.

H-terminated and SiO_x_-grown p-Si (100) wafers were prepared through the treatment of diluted hydrofluoric acid and hot-water-oxidation, respectively, and these wafers were deposited with an amorphous HfO_x_ thin film using the atomic layer deposition (ALD) process^39–41^. The chemical composition of HfO_x_ was confirmed using energy-dispersive X-ray spectroscope (EDXS) mapping and X-ray photoelectron spectroscopy (XPS) spectra (See Supplementary Figs 1 and 2). Figure 1a,b show the high-resolution transmission electron microscope (HRTEM) images for the ALD HfO_x_ thin film deposited on the H-terminated and SiO_x_-grown Si wafers. The HfO_x_ deposited on H-terminated Si was observed to have a 3-nm-thick HfSiO_x_ interfacial layer, which is caused by a diffusion of the Si atoms during the ALD (see Fig. 1a)^42^. In contrast, the interfacial layer was not found in the SiO_x_-grown Si. Figure 1b shows the TEM image for the atomically thin SiO_x_ interlayer inserted between the HfO_x_ and Si without the formation of a HfSiO_x_ layer. This difference is likely due to the bond strength of Si–O (≈190 kcal·mol^−1^)^43^ being higher than that of Si–H (≈80 kcal·mol^−1^)^44^. The stronger Si–O binding formed by SiO_x_ growth prevents the Si diffusion on the Si surface, thereby creating the HfO_x_/SiO_x_ bilayer without formation of HfSiO_x_. The chemical quality of the interface between HfO_x_ and Si is clearly confirmed by the angle-resolved X-ray photoelectron spectroscopy (ARXPS) results. For the Si2p XPS spectra at the low incident angle that represents the interface information, the H-terminated Si wafer has one dominant state of Si^3+^ originating from the HfSiO_x_ interfacial layer (Fig. 1c), whereas the SiO_x_-grown Si wafer has various sub-oxide states of Si^1+^, Si^2+^, Si^3+^, and Si^4+^ (corresponding to Si_2_O, SiO, Si_2_O_3_, and SiO_2_, respectively; see Fig. 1d)^45,46^.

**Figure 2:**
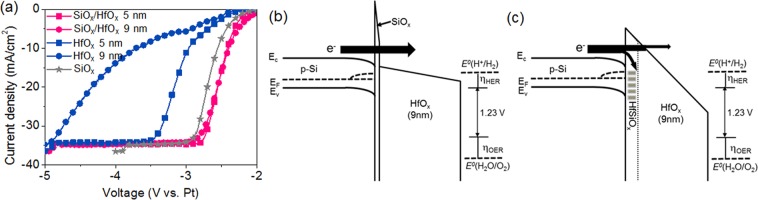
Comparison of PEC performances for p-Si photocathode protected with a-HfO_x_ monolayer and a-HfO_x_/SiO_x_ bilayer. (**a**) LSV curves of HfO_x_ monolayer and HfO_x_/SiO_x_ bilayer protected Si photocathodes. Energy band diagrams of (**b**) HfO_x_ monolayer and (**c**) HfO_x_/SiO_x_ bilayer protected Si photocathodes under application of voltage for the water-splitting reactions.

Linear sweep voltammetry (LSV) responses for the Si photocathodes protected with HfO_x_, SiO_x_, and HfO_x_/SiO_x_ were obtained in 0.5-M H_2_SO_4_ to compare the charge transfer kinetics for the PEC water-splitting reaction (Fig. 2a). Two electrode systems consisting of a Si photocathode and Pt anode were used to determine the potential requirement for the self-biased water-splitting reaction. With a thermodynamic potential barrier of 1.23 eV, additional kinetic overpotential is required for the water-splitting reaction. Owing to the sluggish kinetics of the hydrogen evolution reaction (HER) and oxygen evolution reaction (OER), the application of a minimum potential of ~2.8 V vs. Pt is required for realizing a high photocurrent level of 30 mA·cm^−2^. The deposition of a 5-nm-thick HfO_x_ results in an additional overpotential of 1.1 V at 30 mA·cm^−2^ owing to the tunneling barrier of HfO_x_, and the increase in the thickness to ~9 nm further increases the overpotential by 1.2 V (indicated by blue curves in Fig. 2a). Interestingly, on inserting a thin SiO_x_ interlayer between the HfO_x_ and Si (creating a HfO_x_/SiO_x_ bilayer), the additional overpotential is completely reduced regardless of the HfO_x_ thickness (indicated by the pink curves in Fig. 2a). The same behavior was observed in the dark LSV characteristics for the degenerated doped n-type (n^+^-Si) wafer, which is only attributed to the charge transfer kinetics (Supplementary Fig. 3). This result indicates that the insertion of SiO_x_ makes the charge transport independent of the tunneling barrier of the HfO_x_, which is also verified by the electrochemical impedance spectra (Supplementary Fig. 4).

The negligible tunneling barrier can be explained by the effective engineering of an energy band after the insertion of SiO_x_ under the application of a voltage for driving the PEC water-splitting reaction. When applying potential to the electrolyte/insulator/semiconductor configuration, the electric field can be built up across the insulator, thereby inducing a potential drop at the insulator layers^47^. When the potential is applied across the HfO_x_/SiO_x_ bilayer, the potential drop can be expressed as

1 $${V}_{ox}={E}_{Si{O}_{x}}{t}_{Si{O}_{x}}+{E}_{Hf{O}_{x}}{t}_{Hf{O}_{x}}$$

where E and t are the electric field and thickness, respectively. E_SiOx_ and E_HfOx_ are related as

2 $${E}_{Hf{O}_{x}}=(\frac{{{\rm{\varepsilon }}}_{Si{O}_{x}}}{{{\rm{\varepsilon }}}_{Hf{O}_{x}}}){E}_{Si{O}_{x}}$$

The electric field is dominantly applied in the SiO_x_ layer rather than in HfO_x_ owing to a dielectric constant of SiO_x_ (3.9) being much lower than that of HfO_x_ (11)^47^. This allows a predominant potential drop across the SiO_x_ layer. The potential drop across the SiO_x_ layer under the application of potential results in a lying conduction band of HfO_x_ that is relatively lower than that of Si, as described in Fig. 2b, which shows the band diagram of the HfO_x_/SiO_x_ bilayer. As a result, a tunneling barrier of SiO_x_ only affects the transport of the charged carrier in the HfO_x_/SiO_x_ bilayer, and consequently, the effective tunneling distance becomes equal to the thickness of the SiO_x_ monolayer. In contrast, the potential drop for the HfO_x_ monolayer occurred entirely in the HfO_x_ monolayer, thus creating a triangular barrier as described in Fig. 2c^48^. Because of the triangular barrier, the tunneling resistance is differentiated with the current density. In the case of a low current level (<~10 mA·cm^−2^), the charge carriers are mostly transferred through the upper region of the triangular barrier in order to minimize the tunneling distance and resistance. Consequently, in the low-current level, the overpotential induced by the tunneling resistance is independent of the thickness. However, in the case of a high photocurrent region (>~10 mA·cm^−2^), charge carriers transfer through all the regions of the barrier and suffer from a large tunneling distance and resistance. Therefore, the overpotential in the high-current level is greatly increased with the thickness.

It is also worth noting that the formation of the HfSiO_x_ interfacial layer in the HfO_x_ monolayer induces a hysteresis phenomenon owing to a negative charge trapping/detrapping mechanism at the interfacial layer^49^; the LSV curve is shifted into the anodic direction during the 10 cycles of the LSV scan (see Supplementary Fig. 5). This suggests that the HfSiO_x_ interfacial layer with a high defect density acts as a recombination site for the charge carrier and also causes an increase in the charge transfer resistance. By contrast, the HfO_x_/SiO_x_ bilayer prevented the formation of HfSiO_x_ interfacial layer and the hysteresis. To observe the effect of the SiO_x_ interlayer on the PEC performance of the bilayer protected Si photocathodes, the LSV responses were characterized for the samples with the wet-chemically-grown SiO_x_ interlayer with oxidation times of 10 min (thin-SiO_x_) and 30 min (thick-SiO_x_) and with a thermally grown 1.8-nm-thick SiO_2_ interlayer (Supplementary Fig. 6). A wet-chemically-grown thin-SiO_x_ shows the lowest overpotential as compared to the thicker one and the thermally grown SiO_2_ interlayer.

**Figure 3:**
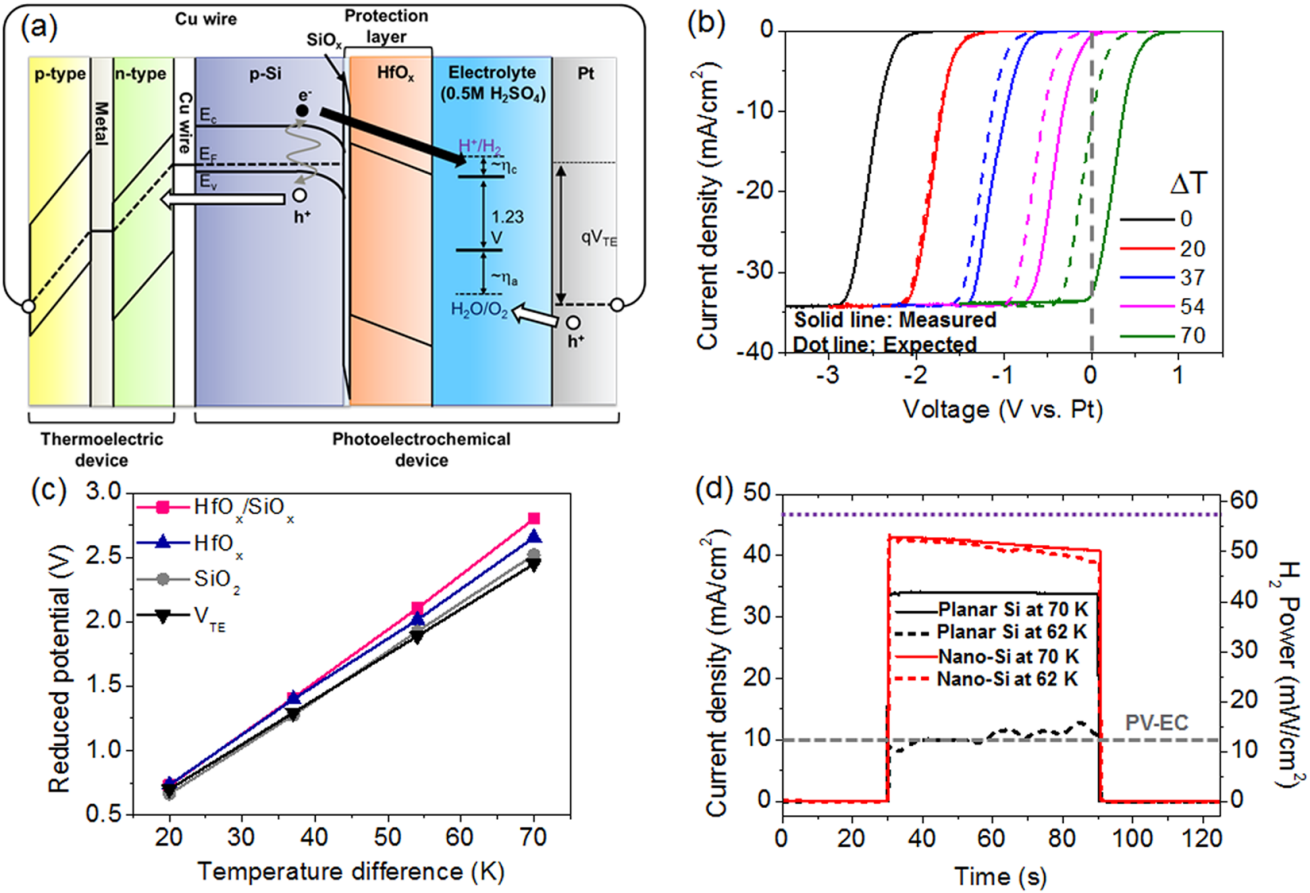
PEC-TE hybrid devices (**a**) Energy band diagram of PEC-TE hybrid device for Seebeck-voltage-driven PEC water-splitting reaction. (**b**) LSV curves of the HfO_x_/SiO_x_ bilayer protected Si photocathode under application of Seebeck voltage as a function of temperature difference (ΔT). (**c**) Plots indicating ΔT-dependent reduced potential for PEC water-splitting reaction; Si photocathodes protected by various insulator layers. (**d**) Photocurrent and P_max_ for PEC-TE device using planar Si and nanostructured Si (nano-Si) at ΔT of 62 K and 70 K. The grey line indicates the P_max_ for the photovoltaic-electrolysis (PV-EC) device^8^.

The engineering of the HfO_x_ barrier height under the application of a voltage gives a further advantage in the PEC-TE hybrid system based on the HfO_x_/SiO_x_ bilayer protected Si photocathode. As demonstrated in previous works, a hybrid system in which a PEC device is electrically combined in series with a TE device could promote a self-biased solar water-splitting reaction. The Seebeck voltage generated in the TE device owing to the thermal gradient (V_TE_) causes the Fermi level of the counter electrodes to be lower than the oxygen evolution level, as depicted in Fig. 3a. This can effectively offset the potential required for driving the self-biased water-splitting reaction while the overall current is determined by the photocurrent of the PEC cell (i.e., the TE is only used as a voltage source)^26^. This was proven by the LSV responses with the driven V_TE_; the LSV curve for the bare Si photocathode (with DHF treatments) was shifted in the anodic direction in proportion to the applied V_TE_ (see Supplementary Fig. 7). Interestingly, in the case of the HfO_x_/SiO_x_ bilayer protected Si photocathode, an anodic shift degree is larger than the applied V_TE_, and the overpotential is further reduced by 350 mV at a 70 °C temperature gradient (see Fig. 3b). To clearly differentiate the shifts, the anodic shift degrees of the LSV curve (reducing overpotential) at a photocurrent of 30 mA·cm^−2^ were plotted as a function of ΔT along with the V_TE_ (Fig. 3c). It should be noted that the slope of the linear plot for V_TE_ is the Seebeck coefficient and is represented by the relation V_TE_ = SΔT. It is apparent that the slop for the HfO_x_/SiO_x_ bilayer protected Si photocathode (41 mV·K^−1^) is much greater than that for V_TE_ (35 mV·K^−1^). For the purpose of comparison, we also characterized the slope for the Si photocathodes grown with monolayers of HfO_x_ and SiO_x_ (see Supplementary Fig. 8 for the LSV curves). The SiO_x_ has a slope (36 mV·K^−1^) that is analogous with that of V_TE_. In contrast, the slope for the HfO_x_ is much greater than that for V_TE_ but smaller than that for the HfO_x_/SiO_x_ bilayer. These results are obtained owing to a different tunneling barrier height and potential drop under the applied V_TE_ (see Supplementary Fig. 9 for the band diagrams).

As a result of applying V_TE_, a saturated photocurrent of 34 mA·cm^−2^ was obtained at a zero potential (i.e., external bias-free). In the PEC-TE hybrid device, an improvement in the photocurrent level is directly correlated with the solar-to-hydrogen conversion efficiency. Thus, we used antireflective Si nanostructures on Si photocathode, which completely absorbed the incident light^50,51^ and greatly improved external quantum efficiency (EQE) for the light-induced charge carrier, maximizing the photocurrent of 42 mA·cm^−2^ (see Fig. 3d and Supplementary Figs 10 and 11). While the PEC-TE hybrid system with planar Si provided the maximum H_2_ power generation (P_Max_) of 42 mW·cm^−2^, the antireflective Si nanostructure photocathode achieved a P_Max_ of 55 mW·cm^−2^, which is four times greater than that of a photovoltaic-electrolysis combination (P_Max_ of ~12 mW·cm^−2^ indicated by the grey line in Fig. 3d).

**Figure 4:**
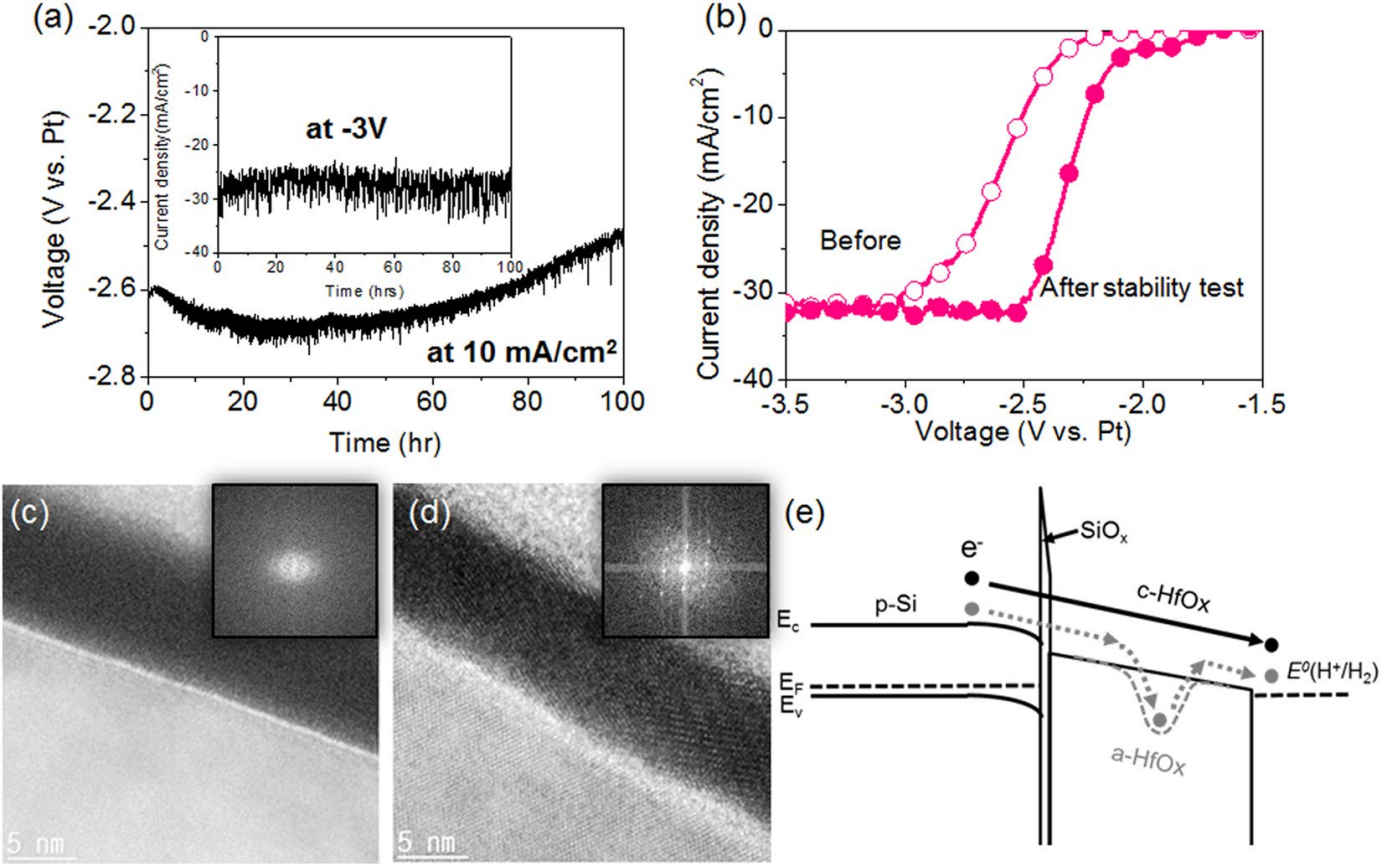
Stability test of HfO_x_/SiO_x_ bilayer protected Si photocathode. (**a**) Measurements of chronopotentiometry at 10 mA cm^−2^ and chronoamperometry at −3 V (inset). (**b**) LSV curves of the Si photocathode before and after the stability test. TEM images of the Si photocathode (**c**) before and (**d**) after the stability test. (**e**) Band diagrams describing the electron transfer kinetics through a-HfO_x_ and c-HfO_x_ tunneling layer.

The long-term stabilities of the HfO_x_/SiO_x_ bilayer protected Si photocathode were performed using chronoamperometry at a photocurrent level of 10 mA cm^−2^ and using chronopotentiometry at an applied potential of −3 V. Due to the HfO_x_ thin film which has an (electro)chemical stability in the acidic electrolyte and transparency for all wavelength region of the incident light, the Si photocathode exhibited a stable water-splitting reaction for over 200 h at high photocurrent level of 33 mA/cm^2^ (Fig. 4a). The stability achieved at high photocurrent by HfO_x_ thin film is the best result compared to other metal oxide thin films (see Supplementary Table 1). TiO_x_ thin films chemically stable in the acidic and alkaline electrolytes have been widely used as a protection layer and have demonstrated long-term stability over 960 hr^52^. However, it has been recently reported ultraviolet (UV) light absorbed in TiO_x_ causes the electrochemical reduction of TiO_x_^53^. Because of this issue, the TiO_x_ protected Si photocathode operates reliably only when there is no UV irradiation, resulting in low photocurrent. Under the stable operation, the H_2_ evolution was characterized by measuring a volume of the H_2_ gas collected under a simulated AM 1.5 G 1-Sun illumination while operating under a certain photocurrent level of 32 mA·cm^−2^ for 50 min (Supplementary Fig. 12). The measured H_2_ volumes were corresponded well with the theoretically calculated values at a 100% Faraday efficiency based on the total charge passed. After the PEC operation for 200 h, interestingly, the potential was finally shifted in the anodic direction by 200 mV, which was identified by the anodic shift of the LSV curve (see Fig. 4b). This was found to be the result of the crystallization of the as-deposited a-HfO_x_ during the PEC water-splitting reaction as observed in the TEM images and X-ray diffraction (XRD) results (Supplementary Fig. 13) of the HfO_x_ layer before and after the 200 h stability test (see Fig. 4c,d). Because of a bulk defect state in the a-HfO_x_ that trapped the charge carrier, a substantial resistance was induced for the charge carrier across the a-HfO_x_, as depicted in Fig. 4e. In contrast, the crystallized HfO_x_ significantly reduced the bulk defect density in the a-HfO_x_ in order to improve the charge transfer kinetics. The crystallization during the solar water splitting reaction at the room temperature condition is an intriguing phenomenon, as it is known to crystallize a-HfO_x_ in the monoclinic phase at temperature of 500 °C^54^. This can be attributed to an electroforming phenomenon wherein the transition metal oxide can be reformed under the electrical field^55^. This has been observed in transition-metal-oxide-based solid-state electronic devices. This finding presents a new method of improving the insulator quality used in PEC devices.

## Discussion

We have presented the PEC-TE hybrid device using HfO_x_/SiO_x_ bilayer protected the Si photocathode in which the HfO_x_ thin film has a strong corrosion-resistance against the acidic electrolyte. The application of the Seebeck voltage generated in the TE device to the PEC device not only drove the self-biased solar water-splitting reaction but also promoted the ease of charge transfer through the HfO_x_ tunneling barrier. On applying the Seeback voltage to the HfO_x_/SiO_x_ bilayer/Si photocathode, the conduction band of HfO_x_ reached an energy level lower than the conduction band of Si, thereby allowing the electron transfer to become independent of the HfO_x_ tunneling barrier. Because of the kinetic benefit, the voltage required for the self-biased water-splitting reaction of the Si photocathode is lowered by 0.35 V. As a result, the planar-Si-photocathode-based PEC-TE hybrid realized the self-biased water-splitting reaction with the evolved H_2_ power of 40 mW cm^−2^ at a temperature difference of 70 °C. By adopting antireflective Si nanostructures that improve the photocurrent level to 42 mA·cm^−2^, the stored H_2_ power reached ~52 mW·cm^−2^. The HfO_x_ thin-film protection ensured long-term stable PEC operation for over 200 h. During the stability test, we observed the phase transformation of HfO_x_ from the amorphous structure into a crystalline structure, which reduces the bulk defect density and thus the kinetic overpotential for the charge transfer through the HfO_x_ layer.

## Method

Preparation of HfO_x_ thin-film protected Si photocathodes. A single-crystalline Si wafer with a resistivity of 1–10 Ω cm and degenerately doped n^+^-type Si (100) wafer with a resistivity of 0.001–0.002 Ω cm were used to characterize the PEC response and charge transfer kinetics, respectively. Prior to the deposition of HfO_x_, the Si wafer was dipped into diluted hydrofluoric acid to form an H-terminated surface while removing the native oxide. For growing the SiO_x_ thin film on the surface of the Si wafer, the H-terminated Si wafer is oxidized through treatment with hot water at 90 °C for 10–30 min. ALD of the HfO_x_ thin film was performed for both the H-terminated and SiO_x_-grown Si wafers to obtain the HfO_x_ monolayer and HfO_x_/SiO_x_ bilayer coated Si wafers, respectively. An HfO_x_ thin film was deposited at 280 °C in a 4-inch traveling-wave type ALD reactor (CN-1 Co.) using Tetrakis (ethylmethylamino) hafnium (TEMAHf) and H_2_O precursors with a carrier gas of high purity N_2_ (99.999%). The TEMAHf, N_2_, and H_2_O were sequentially injected for 2.5, 30, and 1.5 s, respectively.

Characterization of HfO_x_ thin films. A HRTEM (JEOL, JEM-2100F, Japan) equipped with an EDXS operated at an accelerating voltage of 200 kV was used to observe the morphology and elemental composition of the HfO_x_-thin-film-coated Si wafers. The thicknesses of the HfO_x_ and SiO_x_ thin films were measured using spectroscopic ellipsometry (Sopra GES 5E, fitted to a Tauc–Lorentz function using the Cauchey model). The chemical states of the HfO_x_ and SiO_x_ thin films were investigated using ARXPS equipped with a monochromatic Al Kα (1.486 eV) source.

Setup of PEC-TE hybrid devices. The PEC device consists of the Si photocathode and Pt mesh anode for the hydrogen evolution reaction (HER) and oxygen evolution reaction (OER), respectively. The back of the Si photocathode was treated with DHF soaking and subsequent scribing using an In–Ga eutectic alloy (Sigma–Aldrich) to fabricate back contacts with a Cu electrode. PEC-TE hybrid systems were used by electrically connecting the PEC device with a TE device in series; the Si photocathode is connected to an anode of the TE device, and a cathode of the TE device is connected to the Pt anode. TE devices with a Seebeck coefficient of 35 mV·K^−1^ and internal resistances of 2.1 Ω, which are commercially available from Kryotherm (Saint-Petersburg, Russia), were employed.

Characterizations of PEC performances. The PEC properties of the Si photocathodes were measured using a potentiostat (Iviumstat, Eindhoven, Netherlands) with a two-electrode configuration (Si photocathode and Pt mesh anode). The PEC responses were characterized using various methods of LSV, chronopotentiometry, and chronoamperometry in 0.5-M sulfuric acid under a 100-mW·cm^−2^ illumination (Xenon lamp equipped with AM 1.5 G filter) and calibrated by a Si photodiode standard cell (PV Measurements, Inc.). The LSV responses were measured under dark and illuminated conditions at a scan rate of 50 mV·s^−1^. The stability and H_2_ evolution measurements were performed using chronopotentiometry and chronoamperometry. Home-made heating and cooling systems were fabricated to control the temperature and were used for applying a constant temperature difference between the front and back of the TE device. The temperature difference was monitored using K-type thermocouples (Center 306 Data Logger, New Taipei City Taiwan). The quantity of hydrogen evolved at the Si photocathode under illumination and the Seebeck voltage application was measured using the volume displacement method wherein the volume of a water-filled flask changes with the evolution of hydrogen gas. A Pt counter electrode covered with a quartz cylinder and proton exchange membrane was used in order to separately collect hydrogen and oxygen gas. All the PEC-TE hybrid system is shown in Supplementary Fig. 14 as a digital image.

## Supplementary information

Supplementary information

## Data Availability

The data supporting the findings of this study are available within the article and its Supplementary Information files. All other relevant source data are available from the corresponding author upon reasonable request.
