# Supplementary material for: Seebeck-voltage-triggered self-biased photoelectrochemical water splitting using HfOx/SiOx bi-layer protected Si photocathodes
Source: Sci Rep. 2019 Jun 24;9:9132. doi: 10.1038/s41598-019-45672-4 (PMC6591395; doi:10.1038/s41598-019-45672-4)
Supplement: Supplementary file 1 — Supplementary information [file 41598_2019_45672_MOESM1_ESM.docx]

Supplementary Materials for

Seebeck voltage triggered self-biased photoelectrochemical water splitting using HfO_x_/SiO_x_ bi-layer protected Si photocathodes

Jin-Young Jung^1^, Dae Woong Kim^1^, Dong-Hyung Kim^1^, Tae Joo Park^1^, Ralf B. Wehrspohn^2,3^, and Jung-Ho Lee^1,*^

^1^Department of Materials and Chemical Engineering, Hanyang University, 55 Hanyangdaehak-ro, Sangnok-gu, Ansan, Kyeonggi-do 15588, Republic of Korea

^2^Institute of Physics, Martin-Luther-Universität Halle-Wittenberg, Germany

^3^Fraunhofer Institute for Microstructure of Materials and Systems IMWS Walter-Hülse-Strasse 1, D06120 Halle, Germany

*Correspondence to: jungho@hanyang.ac.kr


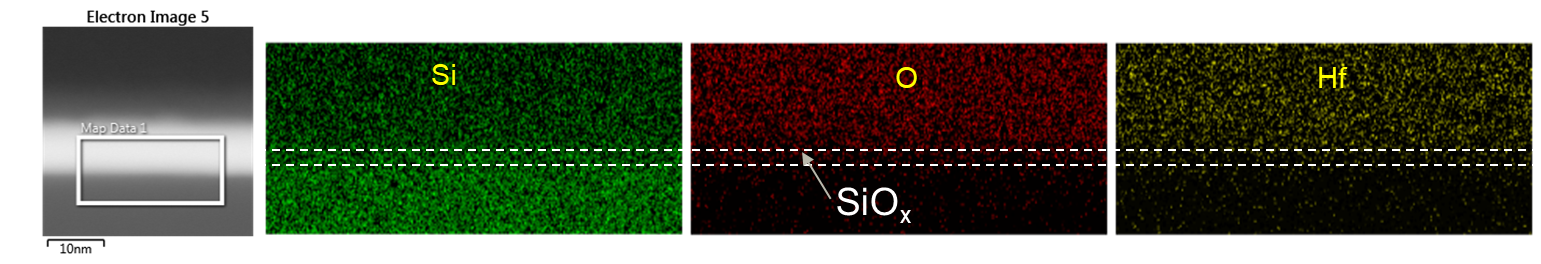


**Supplementary Figure 1.** Results of elemental mapping determined using scanning transmission electron microscopy with EDXS for HfO_x_/SiO_x_ bilayer grown Si wafer.

**
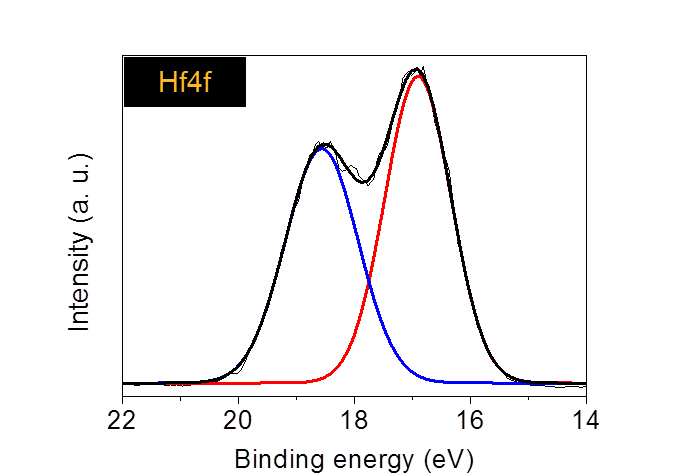
**

**Supplementary Figure 2.** Hf4f XPS spectrum of HfO_x_ thin film.


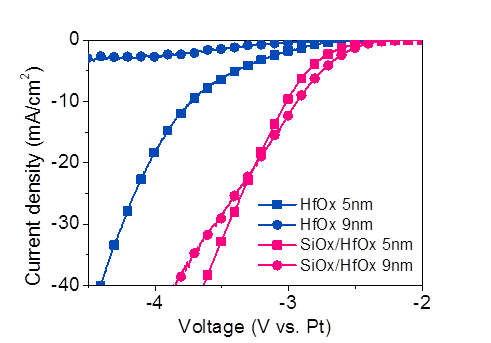


**Supplementary Figure 3.** LSV curves for HfO_x_ monolayer (blue curves) and HfO_x_/SiO_x_ bilayer (pink curves) deposited n^+^-Si electrodes.


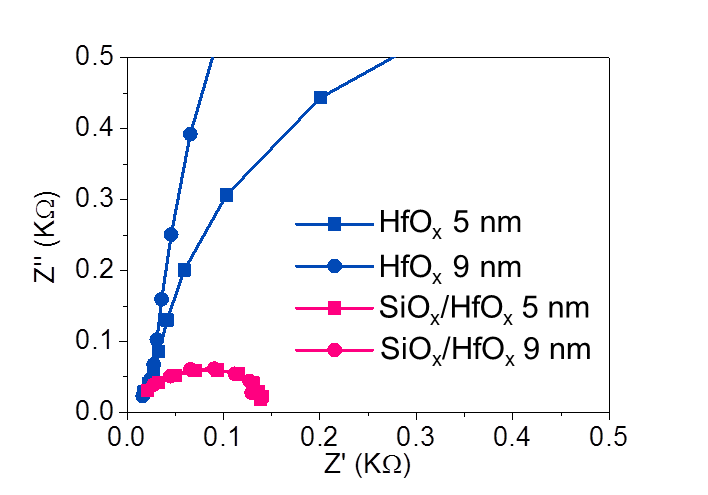


**Supplementary Figure 4.** Nyquist plots of p-Si photocathodes protected with a-HfO_x_ monolayer and a-HfO_x_/SiO_x_ bilayer.


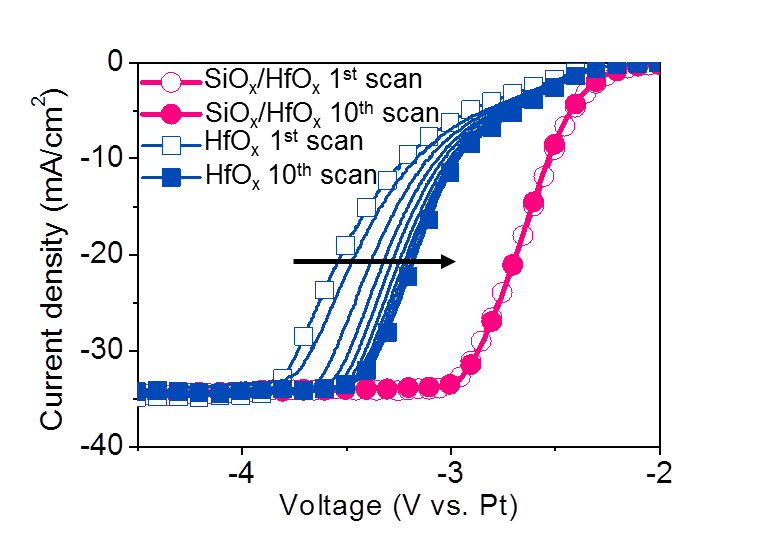


**Supplementary Figure 5.** LSV curves for HfO_x_ monolayer (blue curves) and HfO_x_/SiO_x_ bilayer (pink curves) protected Si photoelectrodes. The arrow indicates the increasing number of scans.


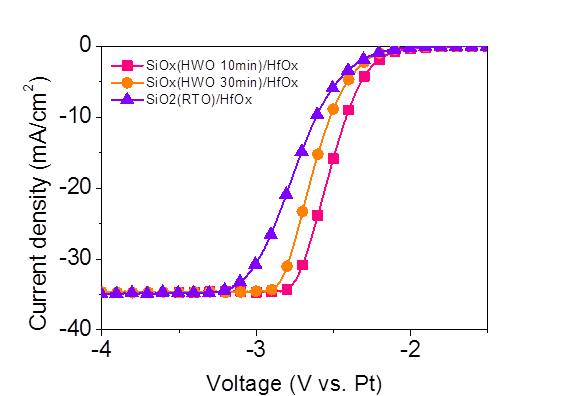


**Supplementary Figure 6.** LSV responses for the Si photoelectrodes with the wet-chemically-grown SiO_x_ interlayer at hot-water-oxidation times of 10 min (thin SiO_x_) and 30 min (thick SiO_x_) and with a thermally grown 1.8-nm-thick SiO_2_ interlayer.


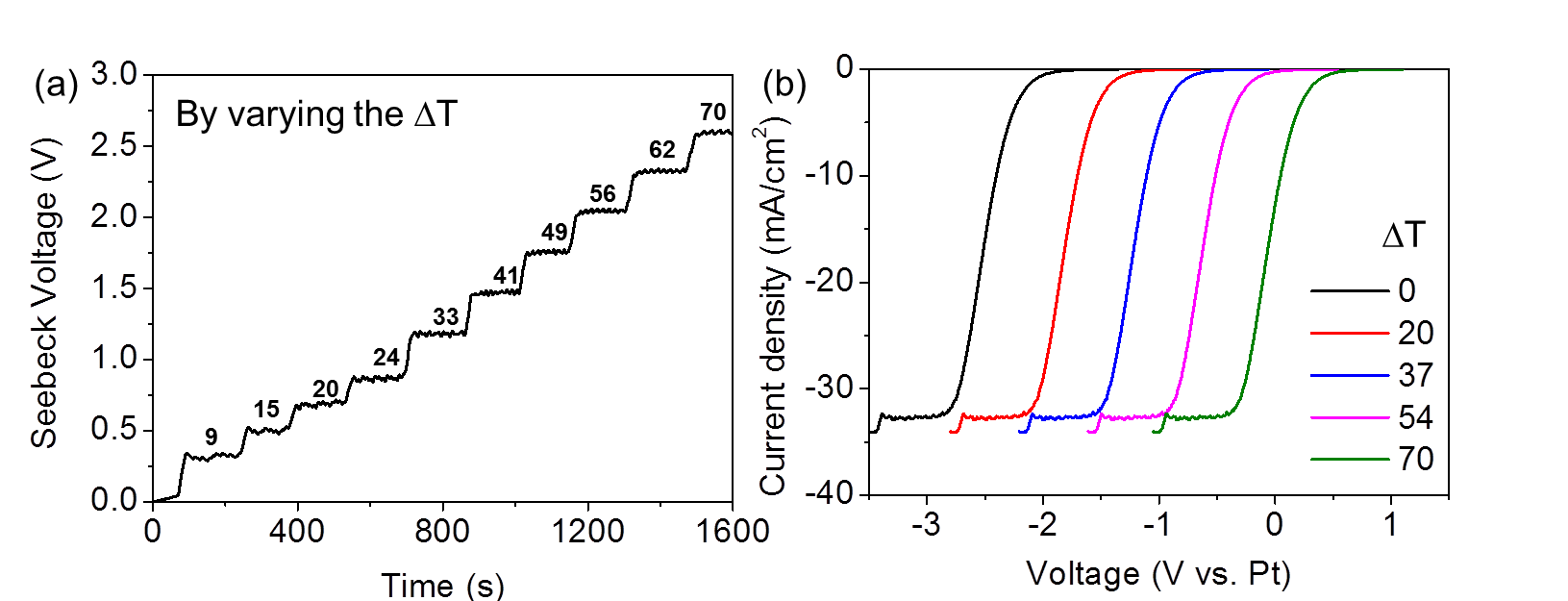


**Supplementary Figure 7.** (a) Seebeck voltage generated in TE (b) LSV curves for the H-terminated Si photocathode obtained on applying Seebeck voltage as a function of ΔT.


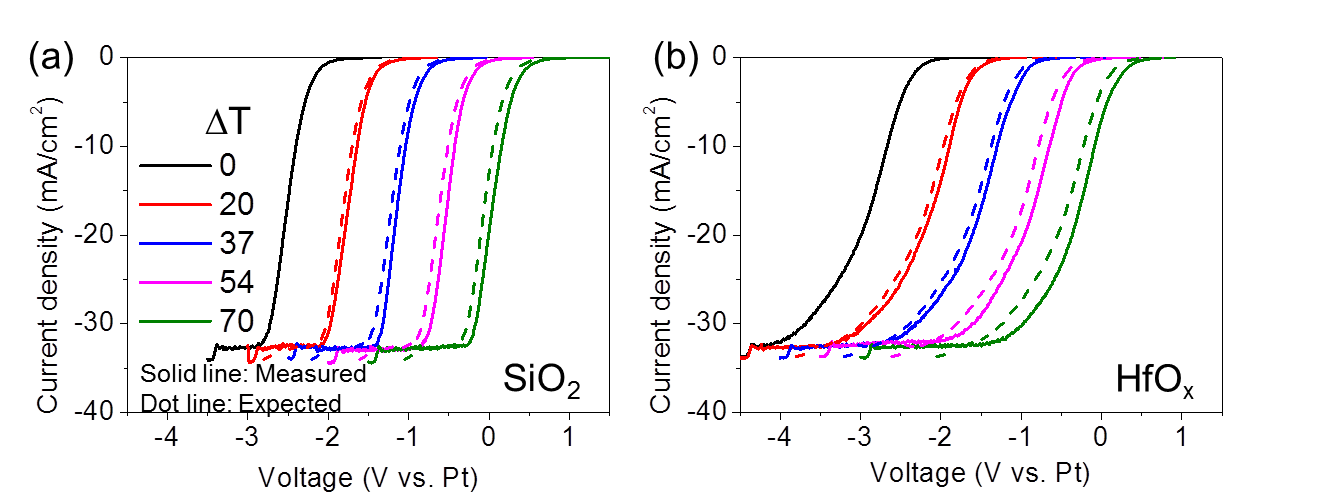


**Supplementary Figure 8.** LSV curves for (a) SiO_2_ and (b) HfO_x_ monolayers protected Si photocathodes obtained on applying Seebeck voltage as a function of ΔT.


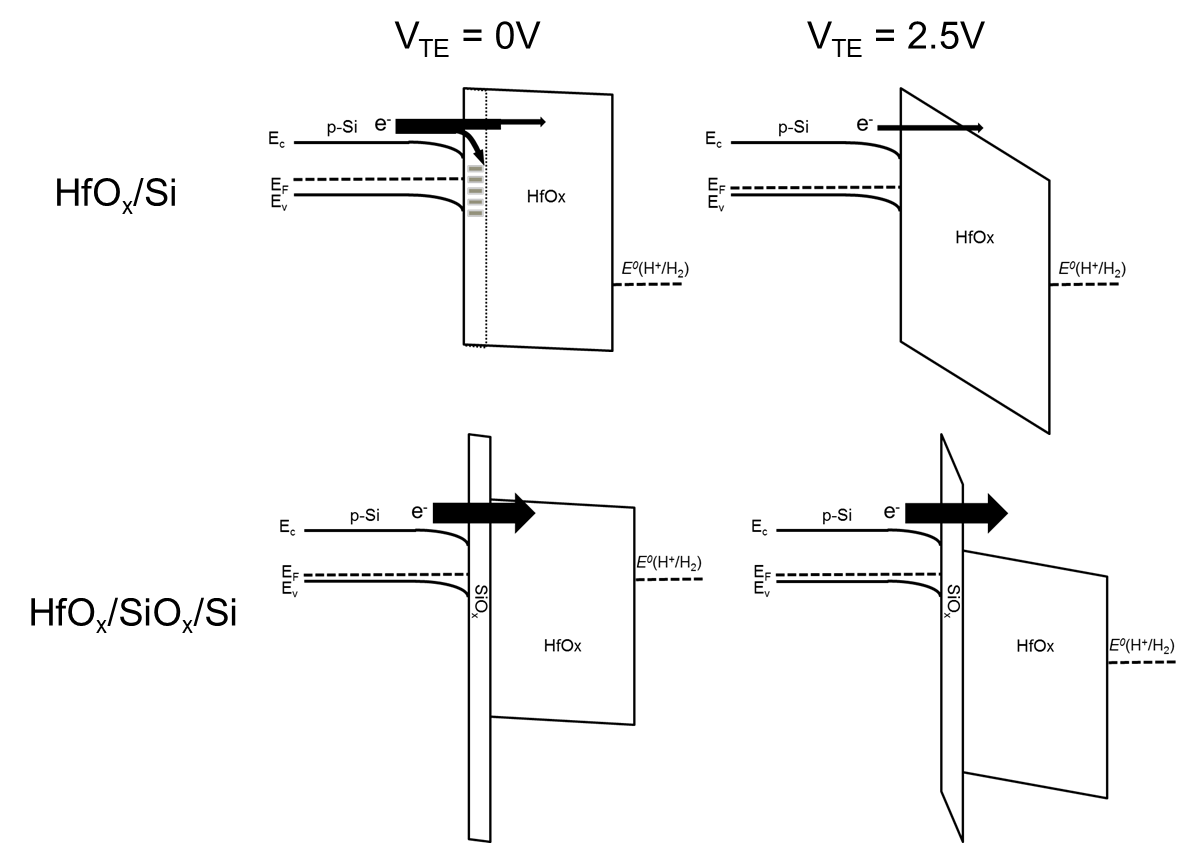


**Supplementary Figure 9.** Band diagrams of the electron transfer kinetics for (top) the HfO_x_ monolayer and (bottom) the HfOx/SiOx bilayer protected Si photocathodes obtained on applying Seebeck voltage of (left) 0 V and (right) 2.5 V.


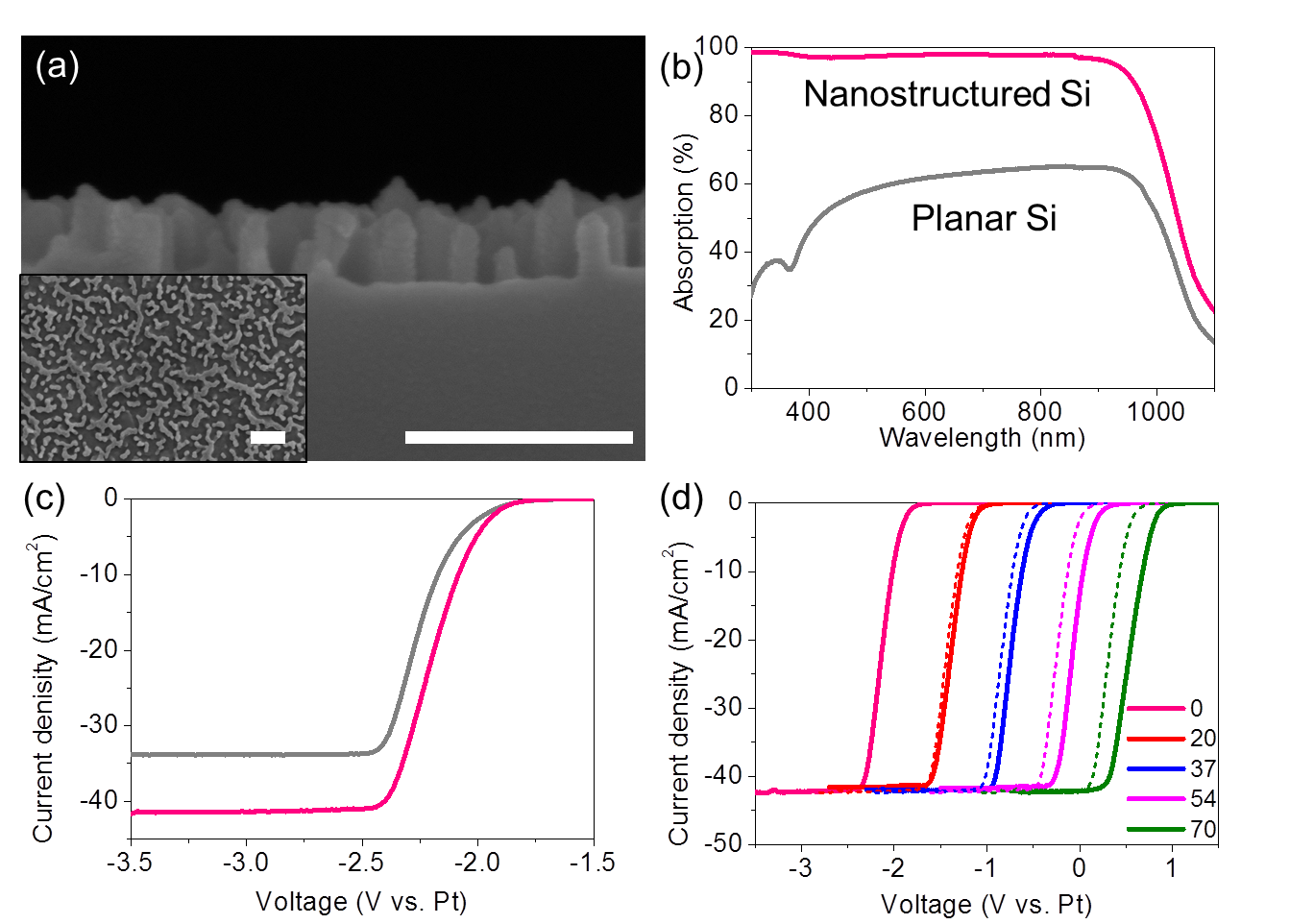


**Supplementary Figure 10.** (a) Cross-sectional and (inset) top-view scanning electron microscopy images of nanostructured Si. (b) Light absorptance spectra and (c) LSV curves of planar Si and nanostructured Si. (d) LSV curves for the nanostructured Si photocathodes obtained on applying Seebeck voltage as a function of ΔT.


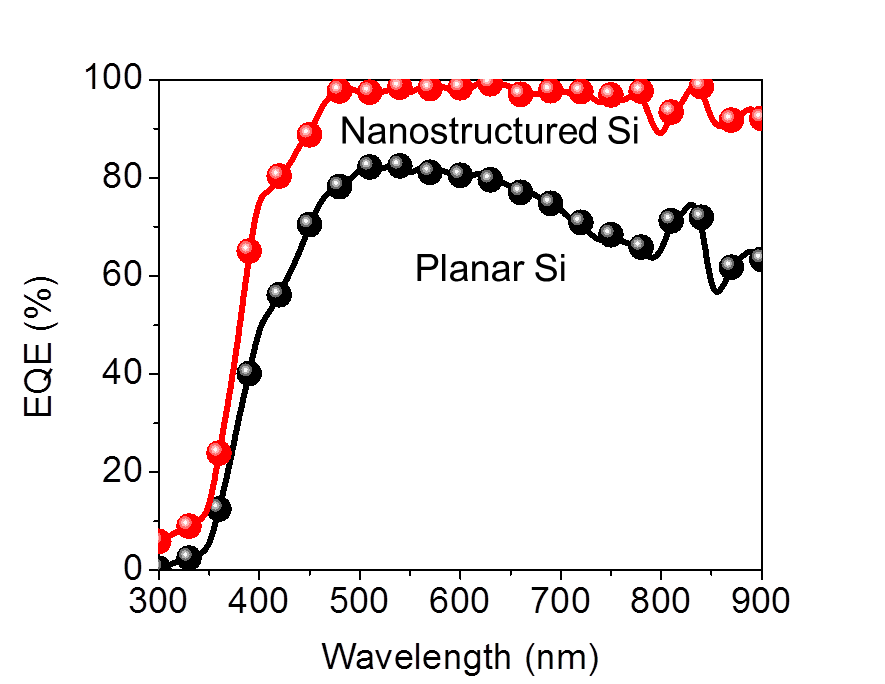


**Supplementary Figure 11.** EQE of planar Si and nanostructured Si.

**Supplementary Table 1.** Representative stability and photocurrent for the Si photocathodes protected with transparent metal oxide thin films.


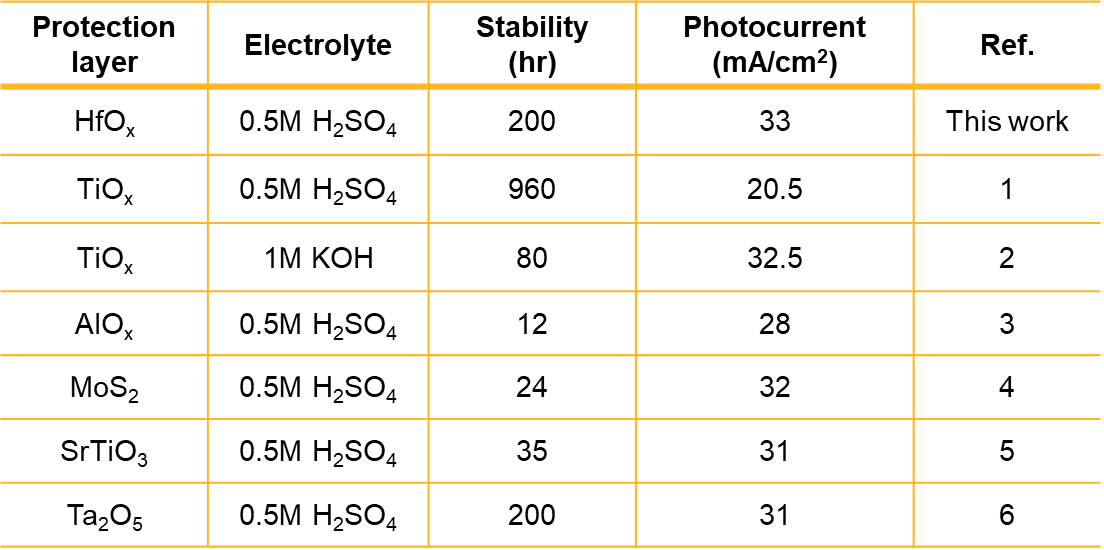


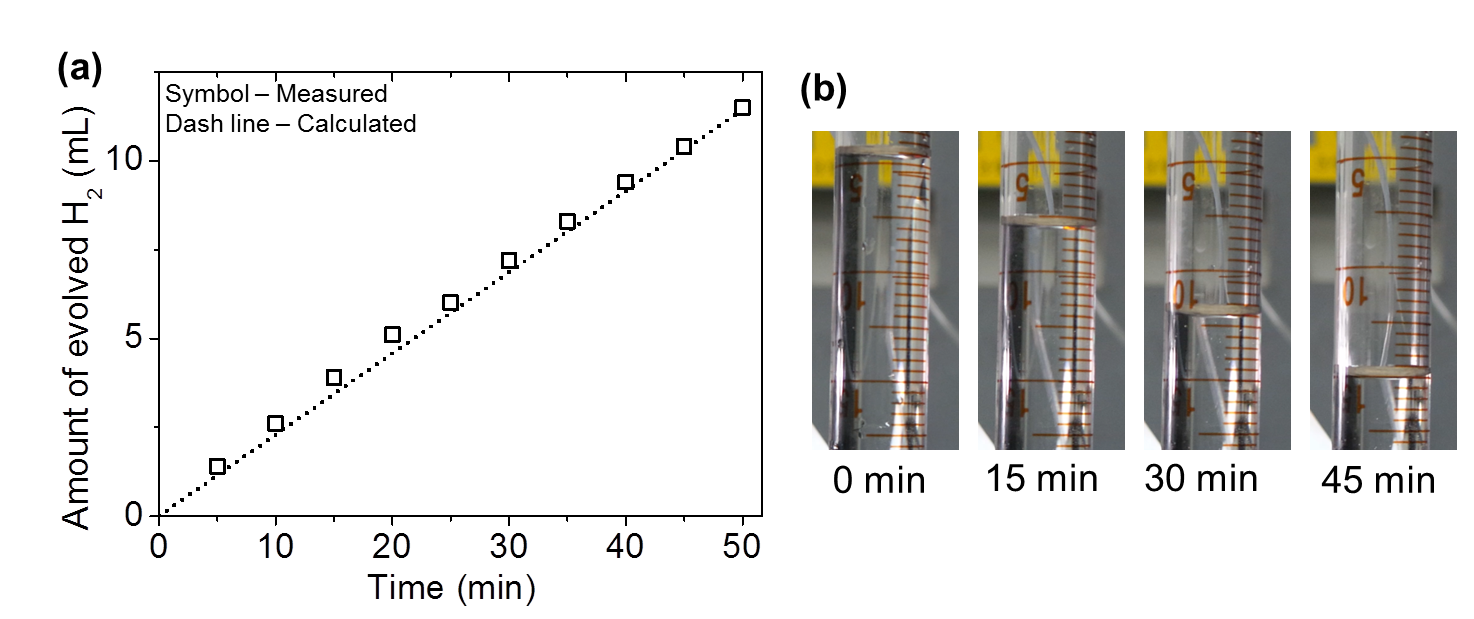


**Supplementary Figure 12.** Hydrogen evolution measured using volume displacement. (a) Plot of amount of H_2_ evolved under the chronopotentiometry measurement at a photocurrent of 32 mA/cm^2^. Symbols (measured) and solid lines (calculated) denote the volumes of collected hydrogen. (b) Digital images of the volume changes corresponding to the measured results of plot (a).


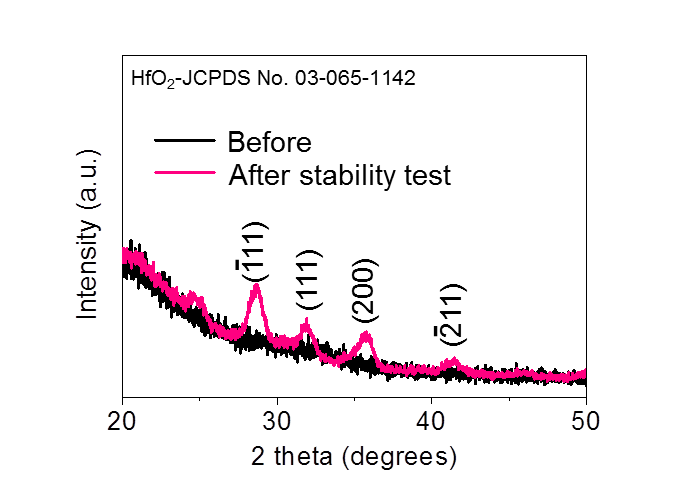


**Supplementary Figure 13.** XRD results of the Si photocathode before and after the stability test.


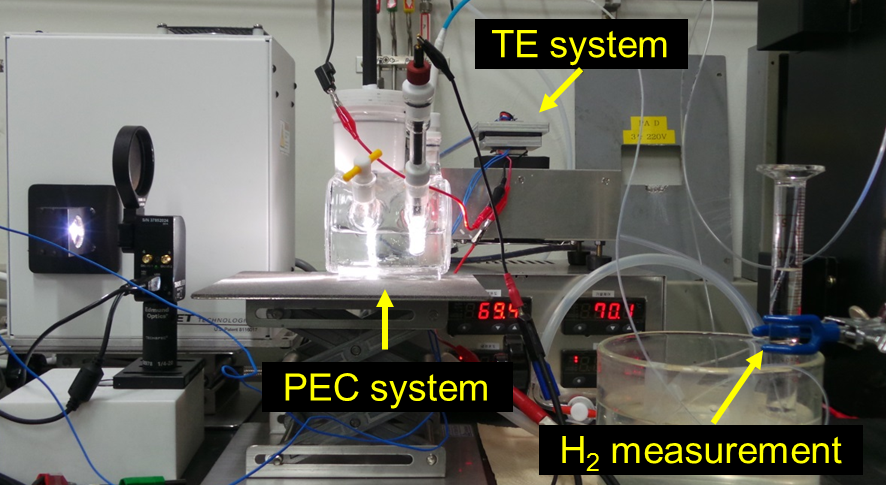


**Supplementary Figure 14.** Digital image of photoelectrochemical system, thermoelectric system, and H_2_ measurement.

**References**

1. Bae, D. *et al*. Carrier-selective p- and n-contacts for efficient and stable photocatalytic water reduction. *Catal*. *Today*, **15**, 59–64(2016).

2. Kast, M. G., Enman, L. J., Gurnon, N. J., Nadarajah, A. & Boettcher, S. W. Solution-deposited F:SnO_2_/TiO_2_ as a base-stable protective layer and antireflective coating for microtextured buried-junction H_2_‑evolving Si photocathodes. *ACS Appl. Mater. Inter.* **6**, 22830–22837 (2014).

3. Choi, M. J. *et al*. Long-term durable silicon photocathode protected by a thin Al_2_O_3_/SiO_x_ layer for photoelectrochemical hydrogen evolution. *J*. *Mater*. *Chem*. *A*, **2**, 2928–2933 (2014).

4. Oh, S., Kim J. B., Song, J. T., Oh, J. & Kim, S. H. Atomic layer deposited molybdenum disulfide on Si photocathodes for highly efficient photoelectrochemical water reduction reaction. . *J*. *Mater*. *Chem*. *A*, **5**, 3304–3310 (2017).

5. Ji, L. *et al*. A silicon-based photocathode for water reduction with an epitaxial SrTiO3 protection layer and a nanostructured catalyst. *Nat*. *Nanotech*. **10**, 84–90 (2015).

6. Wang, T. *et al*. Transparent Ta_2_O_5_ protective layer for stable silicon photocathode under full solar spectrum. *Ind*. *Eng*. *Chem*. *Res*. **58**, 5510–5515 (2019).
